# High-Sensitivity Troponin T as a Predictor of Prolonged Intensive Care Unit Stay and Worse Treatment Outcomes in Patients Undergoing Heart Valve Surgery

**DOI:** 10.3390/jcm14144989

**Published:** 2025-07-15

**Authors:** Piotr Duchnowski, Witold Śmigielski, Piotr Kołsut

**Affiliations:** Cardinal Wyszynski National Institute of Cardiology, 04-628 Warsaw, Poland; wsmigielski@ikard.pl (W.Ś.); pkolsut@ikard.pl (P.K.)

**Keywords:** troponin T, intensive care unit, heart valve surgery, postoperative complications, biomarkers

## Abstract

**Background:** Prolonged stays in the postoperative intensive care unit (ICU) for patients undergoing heart valve surgery are mainly caused by the development of complications. In turn, with the extension of the ICU stay, there is a risk of developing further serious postoperative complications. The main aim of the present study was to evaluate selected biomarkers in terms of their predictive potential for a prolonged postoperative stay in the ICU. **Methods:** This prospective study was conducted on a group of patients undergoing heart valve surgery. The primary endpoint was prolonged postoperative intensive care unit stay beyond 3 days (above the median). Logistic regression analysis was used to assess the predictors of the occurrence of the primary endpoint. **Results:** A total of 631 patients were included in the study. The median stay in the ICU was 3 days (2–5). A total of 265 patients required a prolonged stay in the ICU. In multivariate logistic regression analysis troponin T measured before surgery (*p* = 0.014), EuroSCORE II operative risk score (*p* = 0.004), troponin T measured the day after surgery (*p* = 0.005), preoperative RDW level (*p* = 0.005) and the presence of preoperative atrial fibrillation (*p* = 0.002) were independent predictors of the primary endpoint. **Conclusions:** Patients with elevated troponin T values determined both before the procedure and in the early postoperative period should be given special attention, because this group of patients is burdened with an increased risk of prolonged stay in the postoperative ward, the occurrence of serious postoperative complications and ultimately worse prognosis.

## 1. Introduction

The early postoperative period in patients undergoing cardiac surgery due to valvular heart disease is associated with a need to stay in the intensive care unit (ICU). This is a particularly critical period for the patient’s future fate. Prolonged stay in the postoperative unit is due to, among other factors, the development of complications such as hemodynamic instability, arrhythmias, cardiogenic shock, acute kidney injury, respiratory failure requiring prolonged mechanical ventilation, postoperative bleeding and/or the need for rethoracotomy [1,2,3,4]. In turn, with a prolonged ICU stay, the risk of developing further serious postoperative complications such as infections, thromboembolic complications or the development of pressure ulcers increases significantly. The costs of total hospitalization and the risk of death also increase [5,6,7]. The available literature lacks clear information on predictive factors of prolonged stays in the intensive care unit. In recent years, very important biomarkers of myocardial damage have gained particular importance in various fields of cardiology in the context of diagnosis, treatment and prognosis, i.e., high-sensitivity troponin T (hs-TnT), which is currently among the main tools used in the daily practice of every cardiologist and, increasingly, every cardiac surgeon [8,9,10,11]. The main aim of the presented study was to evaluate selected biomarkers determined in the perioperative period in terms of their predictive potential for prolonged stay in the postoperative ward in patients undergoing cardiac surgery due to valvular heart defects.

## 2. Methods

A prospective study conducted at the Cardinal Stefan Wyszyński National Institute of Cardiology in Warsaw, Poland in 2014–2021 on a group of patients undergoing heart valve surgery. The exclusion criteria for patients included in the study were as follows: lack of consent to participate in the study, age below 18 years, presence of a porcelain aorta, significant atherosclerotic changes in the carotid arteries described in the imaging study, presence of autoimmune diseases, presence of chronic intestinal inflammation and presence of active neoplastic disease. Blood tests to assess morphology and biochemical parameters were performed before the procedure, immediately after the patient’s arrival in the ICU after the procedure, and on the first morning after the procedure. The Troponin hs-STAT test (Roche, Munich, Germany) was used to assess three high-sensitivity plasma troponin T measurements: TnT–troponin T measured before surgery; TnT I–troponin T measured immediately after surgery; and TnT II–troponin T measured the day after surgery. The procedure was performed under general anesthesia with extracorporeal circulation. The primary endpoint was prolonged postoperative intensive care unit stay beyond 3 days (above the median). The observation period of patients included in the study was until discharge from the hospital or until death during their current hospitalization. The consent to conduct the study was given by the local Bioethics Committee at the Cardinal Wyszyński National Institute of Cardiology in Warsaw, study number 2.32/VI/18. Each patient included in the study provided consent by signing the consent form.

### Statistical Analysis

Statistical analyses were performed using the STATISTICA 12 program (StatSoft Polska Sp. z o.o.; Krakow, Poland). Collected data were presented as median (Q1–Q3) and frequency (%). Intergroup cooperation was assessed using the Mann–Whitney U test for quantitative variables and the chi-square test for qualitative variables. When the distribution of analyzed variables differed from the normal distribution, a nonparametric test (Shapiro–Wilk test) was used. Univariate logistic regression analysis was used to assess the predictors of the occurrence of the primary endpoint. Statistically significant variables obtained in univariate analysis were used to perform multivariate logistic regression analysis. A period exceeding the median of this stay was considered as a prolonged period of stay in the ICU. The level of significance was set at *p* < 0.05.

## 3. Results

The study included 631 patients undergoing cardiac surgery for valvular heart disease. The median age of patients included in the study was 65 years (58–71). The median stay in the ICU was 3 days (2–5). The median value of troponin T (TnT) measured before surgery for the entire study group was 12.4 ng/L (7.5–21), the troponin T was measured in the immediate postoperative period (TnT I) as 599 ng/L (36–1018) and in the first postoperative day (TnT II) as 667 ng/L (376–1272). Table 1 presents the characteristics of the patients. A total of 265 patients required a prolonged stay in the ICU. Predictors of the primary endpoint are described in Table 2. In multivariate logistic regression analysis, independent predictors of the primary endpoint were TnT (OR 1.381; 95 CI 1.084–1.759; *p* = 0.014), EuroSCORE II operative risk score (OR 1.141; 95 CI 1.041–1.251; *p* = 0.004), hs-TnT II (OR 1.450; 95 CI 1.116–1.885; *p* = 0.005), preoperative RDW level (OR 1.244; 95 CI 1.043–1.484; *p* = 0.005) and the presence of preoperative atrial fibrillation (OR 1.957; 95 CI 1.261–3.035; *p* = 0.002).

In the group of patients with prolonged ICU stay, all three troponin T measurements were higher, as TnT 15.8 ng/L (10.1–26.7), TnT I 766 ng/L (489–1275) and TnT II 962 ng/L (519–1728), respectively, compared to patients with an ICU stay up to 3 days, TnT 10.8 ng/L (6.6–19), TnT I 485 ng/L (323–779) and TnT II 512 ng/L (335–925) (*p* < 0.001), respectively. Patients with prolonged stay in the postoperative ICU above the median value were characterized by older age, the presence of atrial fibrillation before the procedure and diabetes mellitus, more advanced heart failure according to the NYHA classification and higher values of pre-operative NT-proBNP and a trend towards a higher incidence of coronary artery disease was observed. Moreover, in this group of patients, the occurrence of serious postoperative complications was observed significantly more frequently, such as the need for prolonged administration of inotropic drugs, cardiogenic shock requiring mechanical circulatory support, postoperative CNS stroke, acute kidney injury (AKI), postoperative in-hospital pneumonia, the need for reteracotomy for any reason, multiple organ dysfunction syndrome, sudden cardiac arrest 30-day death and in-hospital mortality.

## 4. Discussion

In the present study, 265, or about 41%, of the patients treated with cardiac surgery for valvular heart disease, required a prolonged (above the median) stay in the intensive care unit. According to the results of the above study, one of the main causes of prolonged stay in the postoperative ward in patients treated with cardiac surgery is postoperative heart failure, which requires, among other factors, the prolonged administration of drugs with positive inotropic effects. This complication occurred in 222 patients, of whom as many as 161 required a prolonged stay in the ICU. Postoperative heart failure, characterized among others by reduced cardiac output, causes reduced tissue perfusion, which in turn promotes the development of dysfunction of individual organs, such as neurological/psychological disorders observed in the ICU, increased renal parameters resulting from AKI or multiple organ dysfunction syndrome. Prolonged stay in the intensive care unit in immobilization conditions also promotes nosocomial infections, such as pneumonia, thromboembolic complications and the development of further adverse effects, including death [5,6,12,13,14,15,16]. Therefore, knowledge of predictive factors for prolonged stay in the postoperative ICU is extremely important. It allows us to pay special attention to patients who are at particularly high risk of postoperative complications.

In recent years, biomarkers of myocardial damage have been used in various areas of cardiology, i.e., hs-TnT is particularly useful in the everyday practice of cardiologists [17,18]. The results of the presented study confirm this trend. The troponin T measurement before the procedure was an independent predictor of prolonged ICU stay. Troponin T (TnT) is a protein that is part of the contractile apparatus of striated muscle. The function of TnT in all types of striated muscle is the same, but the cardiac variant of TnT (cTnT) is different from the TnT found in skeletal muscle. Therefore, cTnT detected in plasma is a highly specific marker of myocardial damage (necrosis). Highly sensitive troponin tests, which have been available for several years, detect troponin levels with a high degree of reliability. In patients with significant valvular heart disease, pressure or volume overload of the heart chambers usually occurs. The myocardial hypertrophy developing in this group of patients is a response to increasing overload. This mechanism initially restores and maintains left ventricular wall tension. However, long-term myocardial strain causes progressive degeneration of cardiomyocytes, as well as the slow development of necrosis and fibrosis. This is due to, among other things, reduced myocardial perfusion, mainly in the endocardial layer of the heart [19,20,21]. According to the results of the present study, it seems that the overloaded heart muscle in patients with valvular heart disease, which is indicated by, among others, an increased preoperative hs-TnT level, is more sensitive to non-physiological perioperative conditions such as cardioplegia, aortic clamping, extracorporeal circulation, blood loss or a number of drugs introduced during this period, including anesthetics. In addition, troponin T determined in the postoperative period will be an indicator of intraoperative myocardial damage according to the analyses performed, and it is also an independent predictor of a prolonged postoperative stay. According to the authors’ previous studies as well as the available literature, troponin T determined in the early postoperative period is a predictor of, among others, postoperative hemodynamic instability requiring prolonged catecholamine administration, as well as cardiogenic shock and death in the group of patients undergoing heart valve surgery and in patients with coronary artery disease undergoing coronary artery bypass grafting [2,18,22].

To sum up, patients with increased troponin T values determined both before the procedure and in the early postoperative period should be patients with special attention, because this group of patients is burdened with an increased risk of prolonging the stay in the postoperative ward, the occurrence of serious postoperative complications and ultimately a worse prognosis. The parameters that are also associated with a higher risk of prolonging the stay in the intensive care unit are an increased preoperative RDW level and the result of the EuroSCORE II surgical risk calculation as well as the presence of atrial fibrillation in the preoperative period.

The presented study has several limitations. Firstly, it is a single-center study. The study involved a limited number of patients and had a short in-hospital observation period. The lack of external validation may limit the generalizability and reliability of the model across different patient populations and clinical settings. Therefore, in future studies, increasing the number of patients included in the study, expanding the number of centers participating in the study and extending the observation period of patients may allow for confirmation of the obtained results.

## 5. Conclusions

This study showed that the parameter of myocardial damage troponin T determined before the surgery as well as on the first day after surgery was an independent predictor of a prolonged stay in the postoperative intensive care unit. Patients requiring prolonged hospitalization in the ICU experience serious postoperative complications significantly more often and have a significantly increased risk of death. Therefore, patients with elevated troponin T values determined both before the procedure and in the early postoperative period should be given special attention.

## Figures and Tables

**Table 1:** Baseline characteristics of the study population (n = 631).

Preoperative Characteristics of Patients	Values All Patients (*n* = 631)	Values Patients with >3-Day Stay in ICU (*n* = 265)	Values Patients with up to 3-Day Stay in ICU (*n* = 366)	*p*-Value
Age, years	65 (58–71)	67 (60–74)	63 (56–69)	<0.001
LV ejection fraction, (%)	60 (52–65)	60 (50–65)	60 (55–65)	0.07
Right ventricular systolic pressure, mm Hg	38 (30–55)	47 (35–60)	34 (30–45)	<0.001
EuroSCORE II, %	2.35 (1.4–3.8)	3.2 (1.87–4.4)	1.88 (1.1–2.9)	<0.001
Atrial fibrillation, *n* (%)	249 (39)	149 (56)	100 (27)	<0.001
Diabetes mellitus, *n* (%)	101 (16)	53 (20)	48 (13)	0.01
Hemoglobin, g/dL	13.7 (12.7–14.7)	13.4 (12.3–14.4)	13.9 (13–14.9)	<0.001
RDW, %	13.8 (13.3–14.6)	14.2 (13.4–15.3)	13.6 (13.2–14.2)	<0.001
GFR, mmol/L	65 (56–80)	60 (49–74)	69 (60–84)	<0.001
TnT, ng/L	12.4 (7.5–21)	15.8 (10.1–26.7)	10.8 (6.6–19)	<0.001
NT-proBNP, pg/mL	880 (321–1961)	1401 (562–3234)	620 (230–1400)	<0.001
CRP, mg/dL	0.23 (0.1–0.46)	0.31 (0.15–0.55)	0.19 (0.1–0.35)	<0.001
Aortic cross-clamp time, min	66 (60–91)	90 (84–128)	63 (60–78)	0.002
Cardiopulmonary bypass time, min	80 (72–108)	114 (84–128)	75 (71–96)	0.005
Postoperative characteristics of patients				
Prolonged administration of inotropic drugs (longer than 48 h), *n* (%)	222 (35)	161 (60)	61 (16)	<0.001
Renal replacement therapy, *n* (%)	50 (7)	43 (16)	7 (2)	<0.001
Postoperative stroke, *n* (%)	21 (3)	20 (7)	1 (0.2)	<0.001
Postoperative hospital-acquired pneumonia, *n* (%)	40 (6)	37 (14)	3 (1)	<0.001
Re-intubation, *n* (%)	82 (13)	76 (28)	6 (1.6)	<0.001
Rethoracotomy, *n* (%)	78 (12)	65 (24)	13 (3)	<0.001
Mechanical circulatory support, *n* (%)	31 (4)	30 (11)	1 (0.2)	<0.001
Multiple organ dysfunction syndrome, *n* (%)	43 (6)	38 (14)	5 (1.5)	<0.001
Hospital stay after surgery, days	10 (8–16)	12 (919)	8 (7–13)	<0.001
30-day mortality, *n* (%)	26 (4.1)	15 (5.6)	11 (3)	<0.001
In-hospital mortality, *n* (%)	39 (6.1)	33 (12.4)	6 (1.6)	<0.001
TnT I, ng/L	599 (360–1018)	766 (489–1275)	495 (323–779)	<0.001
TnT II, ng/L	667 (376–1272)	962 (519–1728)	512 (335–925)	<0.001
Main procedures				
AVR, *n* (%)	323 (51)	100 (37)	223 (60)	<0.001
AVP, *n* (%)	12 (2)	4 (1.5)	8 (2)	0.41
AVR + MVR, *n* (%)	54 (8)	31 (12)	23 (6)	0.01
AVR + MVP, *n* (%)	12 (2)	7 (2.6)	5 (1)	0.25
AVP + MVP, *n* (%)	2 (0.2)	1 (0.3)	1 (0.2)	0.82
MVP, *n* (%)	110 (17)	53 (20)	57 (15)	0.16
MVR, *n* (%)	109 (7)	64 (24)	45 (12)	<0.001
TVR, *n* (%)	7 (1)	4 (1.5)	3 (1)	0.22
Concomitant procedure				
TVP, *n* (%)	171 (27)	108 (40)	63 (17)	<0.001
CABG, *n* (%)	80 (12)	42 (15)	38 (10)	0.04

Data were characterized by median (Q1–Q3) and frequency (%). Abbreviations: AVP = aortic valve plasty, AVR = aortic valve replacement, CABG = coronary artery bypass graft, CRP = C-reactive protein, GFR = glomerular filtration rate, LV = left ventricle, ICU = intensive care unit, MVP = mitral valve plasty, MVR = mitral valve replacement, NT-proBNP = n-terminal of the prohormone brain natriuretic peptide, RDW = red cell distribution width TnT = preoperative troponin T, TnT I = troponin T measured in 0 day after surgery, TnT II = troponin T measured in the first day after surgery, TVP = tricuspid valve plasty, TVR = tricuspid valve replacement.

**Table 2:** Analysis of predictive factors for the occurrence of the primary endpoint.

	Univariate Analysis			Multivariate Analysis		
Variable	Odds Ratio	95% Cl	*p*-Value	Odds Ratio	95% Cl	*p*-Value
Age, years	1.032	1.017–1.047	<0.001			
Atrial fibrillation, *n* (%)	3.425	2.448–4.791	<0.001	1.957	1.261–3.035	0.002
TnT, ng/L	1.749	1.409–2.172	<0.001	1.381	1.084–1.759	0.009
NT-proBNP, pg/mL	1.673	1.442–1.941	<0.001			
CRP, mg/dL	1.577	1.313–1.894	<0.001			
EuroSCORE II, %	1.308	1.204–1.421	<0.001	1.141	1.041–1.251	0.004
LV ejection fraction, %	0.983	0.968–0.997	0.02			
Right ventricular systolic pressure, mm Hg	1.033	1.21–1.046	<0.001			
Diabetes mellitus, *n* (%)	1.680	1.190–2.584	0.01			
Hemoglobin level, g/dL	0.788	0.710–0.874	<0.001			
RDW, %	1.605	1.394–1.849	<0.001	1.244	1.043–1.484	0.01
GFR, mL/min/1.73 m^2^), *n* (%)	0.970	0.960–0.980	<0.001			
Aortic cross-clamp time, min	1.061	1.012–1.112	0.01			
Cardiopulmonary bypass time, min	1.041	1.006–1.077	0.02			
TnT I, ng/L	2.155	1.704–2.726	<0.001			
TnT II, ng/L	2.185	1.753–2.723	<0.001	1.450	1.116–1.885	0.005

Abbreviations: CRP = C-reactive protein, GFR = glomerular filtration rate, LV = left ventricle, NT-proBNP = n-terminal of the prohormone brain natriuretic peptide, RDW = red cell distribution width TnT = high-sensitivity troponin T, TnT I = high-sensitivity troponin T measured immediately after surgery, TnT II = high-sensitivity troponin T measured one day after surgery.

## Data Availability

The original contributions presented in this study are included in the article. Further inquiries can be directed to the corresponding author.
